# Automatic and Accurate Sleep Stage Classification via a Convolutional Deep Neural Network and Nanomembrane Electrodes

**DOI:** 10.3390/bios12030155

**Published:** 2022-03-02

**Authors:** Kangkyu Kwon, Shinjae Kwon, Woon-Hong Yeo

**Affiliations:** 1 School of Electrical and Computer Engineering, Georgia Institute of Technology, Atlanta, GA 30332, USA; kkwon49@gatech.edu; 2 IEN Center for Human-Centric Interfaces and Engineering, Institute for Electronics and Nanotechnology, Georgia Institute of Technology, Atlanta, GA 30332, USA; skwon64@gatech.edu; 3 George W. Woodruff School of Mechanical Engineering, Georgia Institute of Technology, Atlanta, GA 30332, USA; 4 Wallace H. Coulter Department of Biomedical Engineering, Parker H. Petit Institute for Bioengineering and Biosciences, Georgia Institute of Technology, Atlanta, GA 30332, USA; 5 Neural Engineering Center, Institute for Materials, Institute for Robotics and Intelligent Machines, Georgia Institute of Technology, Atlanta, GA 30332, USA

**Keywords:** automatic sleep stage classification, convolutional neural network, nanomembrane electrode, multi-taper spectrogram

## Abstract

Sleep stage classification is an essential process of diagnosing sleep disorders and related diseases. Automatic sleep stage classification using machine learning has been widely studied due to its higher efficiency compared with manual scoring. Typically, a few polysomnography data are selected as input signals, and human experts label the corresponding sleep stages manually. However, the manual process includes human error and inconsistency in the scoring and stage classification. Here, we present a convolutional neural network (CNN)-based classification method that offers highly accurate, automatic sleep stage detection, validated by a public dataset and new data measured by wearable nanomembrane dry electrodes. First, our study makes a training and validation model using a public dataset with two brain signal and two eye signal channels. Then, we validate this model with a new dataset measured by a set of nanomembrane electrodes. The result of the automatic sleep stage classification shows that our CNN model with multi-taper spectrogram pre-processing achieved 88.85% training accuracy on the validation dataset and 81.52% prediction accuracy on our laboratory dataset. These results validate the reliability of our classification method on the standard polysomnography dataset and the transferability of our CNN model for other datasets measured with the wearable electrodes.

## 1. Introduction

An accurate sleep stage classification [1,2,3] plays a significant role in sleep quality monitoring and the diagnosis of disorders. The polysomnogram (PSG) is widely used in the diagnosis of obstructive sleep apnea (OSA) syndrome [4]. PSG is non-invasive and consists of a simultaneous recording of multiple physiological parameters related to sleep and sleep disorders. Standard polysomnography includes the measurement of various physiological signals such as an electroencephalogram (EEG), an electrooculogram (EOG), an electromyogram (EMG), and an electrocardiogram (ECG). Typically, a series of polysomnographic signals for a 30-second-long epoch is labeled as a certain sleep stage by an expert sleep scorer.

Compared to the manual scoring of sleep stages, automatic sleep stage classification serves as a more efficient way to evaluate a large amount of sleep data. Machine learning algorithms have been adopted in automatic sleep stage classification to increase classification efficiency and performance in recent years. Among them, conventional statistical machine learning algorithms, such as Support Vector Machine [1,5], Hidden Markov Model [6], k-nearest neighbors [7], and Random Forests [7] are adopted at the early stage. Recent progress of deep learning in computer vision, natural language processing, and robotics has advanced these methods to application in automatic sleep stage classification. A convolutional neural network (CNN) has been frequently employed for the task. The weight sharing mechanism at the convolutional layers forces the shift-invariance of the learned features and greatly reduces the model’s complexity, consequently improving the model’s generalization. Other network variants, such as Deep Belief Networks (DBNs), Auto-encoder, and Deep Neural Networks (DNNs), have also been explored. Moreover, Recurrent Neural Networks (RNNs), e.g., Long Short-Term Memory (LSTM), capable of sequential modeling, have been found to be efficient in capturing long-term sleep stage transitions. However, these methods still have limited accuracy in sleep stage classification as they have been trained and tested merely on a public dataset, without validation study with real lab datasets.

This work presents an automatic sleep stage classification model that could be applied to the public dataset, and that could also be applied to the classification of our laboratory datasets measured with a novel wearable system. In the public dataset from ISRUC used for this study [8], four channels of signals (two EEG and two EOG) were selected based on their proximity to the electrode locations of our new wearable system being tested. The four signals were preprocessed with various filters and a multi-taper spectral analysis was performed. They were then split into 30-second-long epochs and converted into spectrogram images to be used and tested with our newly developed CNN sleep stage classification model. This CNN model trained with a public dataset was then applied to our lab dataset measured with nanomembrane electrodes that were pre-processed with the same methods. A comparison study with a multi-taper spectrogram and band-pass-filtered raw signals showed the advantage of a multi-taper spectrogram in enhancing the transferability of the model. The final result of this study supports not only the performance of our new classification model on the standard PSG dataset, but also the model’s transferability to a dataset measured with our novel system.

## 2. Materials and Methods

### 2.1. ISRUC Public Dataset

**Figure 1:**
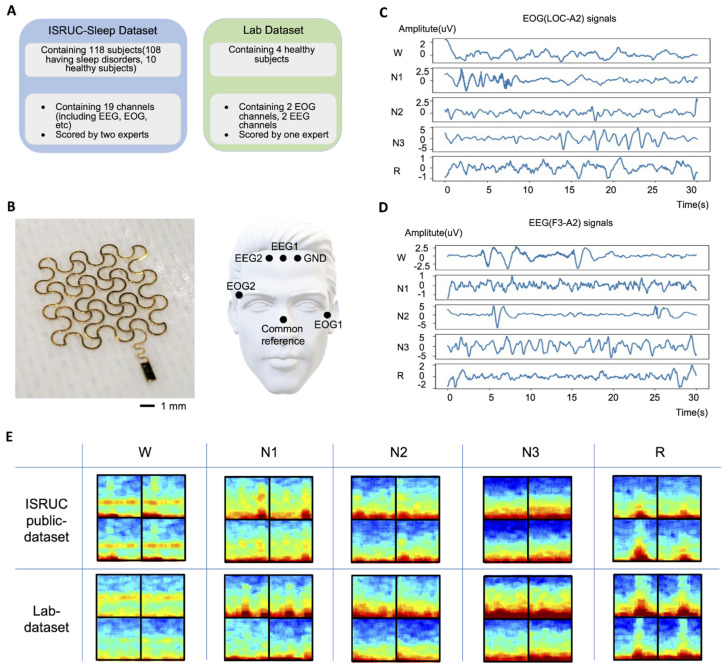
Overview of a public dataset (ISRUC) and the measured lab dataset. (**A**) Detailed information of both datasets. (**B**) Data recording system using nanomembrane bioelectrodes (left) and the sensor mounting locations (right) were the upper center of the forehead (EEG1 and EEG2) to measure two-channel EEG, one electrode on the lower-left corner of the left eye (EOG1) to measure two-channel EOG, and another electrode on the upper-right corner of the right eye (EOG2). (**C**) Measured EOG signals of five different sleep stages: W, N1, N2, N3, and R. (**D**) Measured EEG signals of five different sleep stages: W, N1, N2, N3, and R. (**E**) Examples of multi-taper spectrograms of both the ISRUC public and the lab datasets with five sleep stages. From top-to-bottom and left-to-right, the spectrograms show channels F3-A2, F4-A1, EOGL, and EOGR of the public dataset, and channels EEG1, EEG2, EOG1, and EOG2 of the lab dataset.

The ISRUC sleep dataset (Figure 1A) consists of complete overnight standard PSG recordings of 118 subjects with three health statuses (healthy, sick, and under-treatment). For this study, a total of 100 data from 100 subjects from subgroup 1 were used. ISRUC subgroup 1 included subjects aged between 20 and 85 years (51 on average), with 55 males and 45 females. The subjects were diagnosed with various sleep disorders. More details of individual subject information can be found in Khalighi et al. [8]. Each recording contained six EEG channels (i.e., C3-A2, C4-A1, F3-A2, O1-A2, O2-A1, and F4-A1), two EOG channels (i.e., LOC-A2 and ROC-A1), and three EMG channels (i.e., X1, X2, and X3) as well as an annotation file with detailed events. For this study, two of the EEG channels (F3-A2 and F4-A1) and both of the EOG channels (LOC-A2 and ROC-A1) were used. The recording rate was 200 Hz. In addition, each 30-second-long epoch was labeled with one of the five sleep stages (W, N1, N2, N3, R), as scored by two experts according to the American Academy of Sleep Medicine (AASM) rules [8].

### 2.2. Measured Lab Dataset

The lab dataset used for this study was measured from four healthy male subjects aged between 24 and 27 years. A total of four data were collected, with one recording per subject. To measure our own dataset in the lab, we fabricated a set of nanomembrane, stretchable electrodes using a gold and polyimide (PI) composite, laminated on a silicone adhesive (Figure 1B). This fabrication utilized a microfabrication technique, including photolithography, developing, and etching for making stretchable patterns [9,10]. Afterward, the patterned electrode was transfer-printed onto a soft silicone elastomer for skin mounting [11,12]. For the two-channel EEG setup, two electrodes were placed near the upper center of the forehead (EEG1 and EEG2) to measure frontopolar EEG. For the two-channel EOG, one electrode was placed on the lower-left corner of the left eye (EOG1), and the other electrode was placed on the upper right corner of the right eye (EOG2). All the EEG and EOG channels were derived with a single common reference electrode placed on the bony area of the nose. In PSG, the mastoid is the most widely used common reference point, but the nose is also considered one of the inactive areas suitable as a common reference alternative to the mastoid. Some of the previous works related to EEG measurement adopted the nose as the reference on special occasions [13,14,15,16]. The nanomembrane dry electrode needs to be placed on clean skin without hair to measure signals with high quality, so the nose was selected as the common reference for this study. The ground electrode was placed on the forehead next to the EEG1 electrode. These electrode locations were chosen to develop a compact facial patch device for sleep monitoring in future studies.

A customized printed circuit board (PCB) with an nRF52 (Nordic Semiconductor, Trondheim, Norway) and an ADS 1299 (Texas Instruments, Dallas, TX, USA) was used to collect EEG and EOG signals at a sampling rate of 250 Hz and to transmit them to an Android mobile device via Bluetooth for data storage. The systems used to collect the lab datasets, the nanomembrane-based electrodes and the custom PCB with the nRF52 and ADS 1299 have been extensively studied and validated by comparison with well-established measurement systems by numerous related previous studies [17,18,19,20,21]. A set of example datasets in Figure 1C,D, measured by standard PSG setup and the wearable device, show EOG signals and EEG signals with different patterns based on sleep stages. Figure 1E shows representative multi-taper spectrograms of data measured by the standard PSG setup and the setup used for this study at each of the five sleep stages.

### 2.3. Data Pre-Processing

Rather than training an automatic classification model based on raw sleep data, the data pre-processing method was applied for better classification performance. In the ISRUC public dataset, preprocessing was already applied to eliminate undesired noise and DC offset, enhancing the signal quality and the signal-to-noise ratio. The filtering stage consisted of a notch filter to eliminate the 50 Hz powerline noise and a bandpass Butterworth filter with a lower cutoff of 0.3 Hz and a higher cutoff of 35 Hz for both the EEG and EOG channels. To maximize the performance and transferability of the classification model, the same bandpass filtering parameters were used for the lab dataset. The notch filter setting was adjusted to remove a 60 Hz, rather than 50 Hz, power line noise because the frequency of powerline noise varies based on the place of measurement. Moreover, to match the per-epoch data size to the public dataset, the lab dataset was down-sampled from 250 Hz to 200 Hz by interpolating the datapoints that matched the timepoints corresponding with the 200 Hz sampling rate.

### 2.4. Input Dataset for Deep Learning

**Figure 2:**
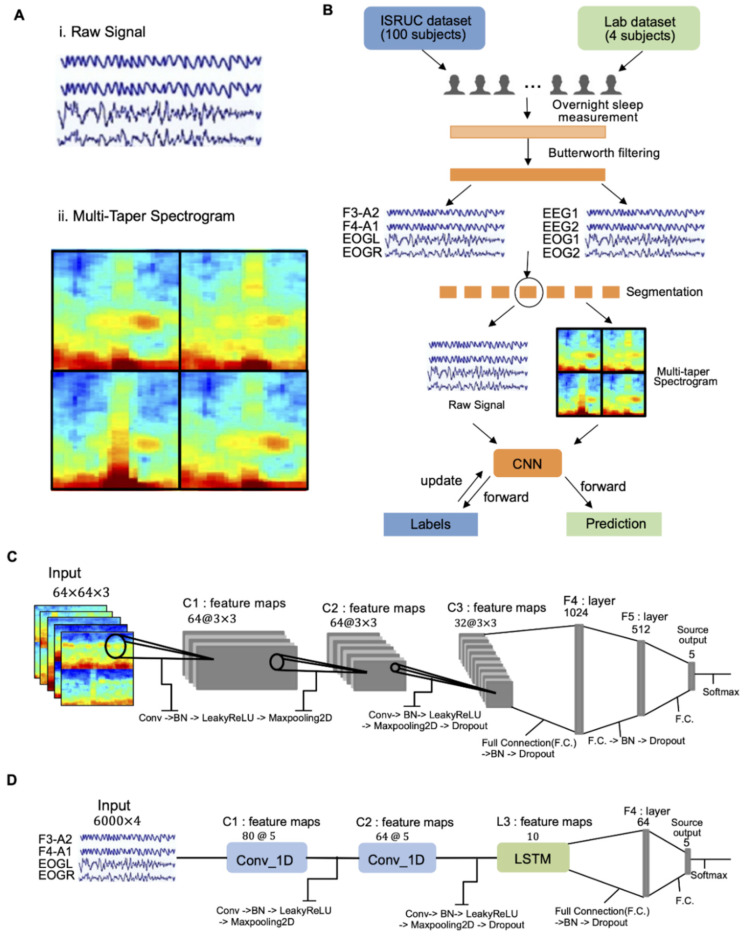
(**A**) Arrangement of four multi-taper spectrograms for deep learning dataset input. (**B**) Flow chart capturing data processing overview. (**C**,**D**) Proposed machine learning architectures for multi-taper spectrograms (**C**) and raw signals (**D**); in this figure, Conv: convolution, F.C.: fully connected layers, and BN: batch normalization.

EEG and EOG signals are commonly analyzed with time-frequency processing techniques or spectrograms, since they are frequently related to behavioral patterns [22]. CNN models are mainly applied to classify and recognize two-dimensional images due to their good compatibility and unprecedented ability to extract image features. As a result, there were many attempts to use spectrograms generated from EEG and EOG signals as the input dataset of a CNN [23,24,25,26]. The spectrogram used for this study was generated by multi-taper spectral analysis, which utilizes multiple taper functions to compute single-taper spectra for better resolution and reduced bias and variance compared to the traditional method. The default settings and parameters provided by Prerau et al. were used for generating spectrograms [27]. The frequency range of spectral analysis was set between 0 and 20 Hz. The time-half-bandwidth product was set to 5, and the number of tapers was set to 9. A window size of 5 s was used, with a step size of 1 s. The entire dataset was first converted into a multi-taper spectrogram, which was then segmented into a 30-second-long epoch. The size of the spectrogram matrix was 30 × 103 for each epoch. Since four data channels were used for this study, four spectrogram matrices were put together as a 60 × 206 matrix, which was then converted into a PNG image file sized 256 × 256 (Figure 2A). The same dataset was prepared with raw data for a comparison study to show the advantage of using a multi-taper spectrogram. The dataset with raw data was composed of bandpass-filtered raw data. The filtered data were then segmented into 30-second-long epochs of four channels, with a matrix size of 4 × 6000. Figure 2B is the flow diagram of how our training dataset (sampled from the ISRUC dataset) and testing dataset (sampled from the lab dataset) were processed.

### 2.5. CNN-Based Classifier

The architecture was developed by trial and error, drawing influence from earlier models [28,29]. Two models were created according to the form of input data: the CNN architecture in the case of image-based multi-taper spectrograms and the CNN + LSTM architecture in the case of time-based raw signal data. The sleep stage classification CNN architecture is described in Figure 2C,D. For the multi-taper spectrogram, the inputs of our CNN were 30-second-long spectrogram images (256 × 256 pixels) of 4 channels (two EEG and two EOG) connected together in a square. The spectrogram image was then resized to 64 × 64 and converted to a value between 0–1 using normalization. Since the color-image consisted of 3 channels (Red, Green, and Blue), every input matrix became a 3-dimensional matrix (64 × 64 × 3). The non-linear activation function employed was the Leaky Rectified Linear Unit (Leaky ReLU). ADAM (learning rate = 0.002) was utilized for the optimization of the CNN architecture. The batch size was set to 16 and the dropout deactivation rate was set to 0.5. Early stopping was used to prevent overfitting by randomly eliminating 20% of the data from the training set and utilizing it as a validation set at the start of the optimization phase. When the validation loss stopped improving, learning rate annealing was performed with a factor of 5. The training was terminated when two successive decays occurred with no network performance improvement on the validation set. A single convolutional cell (Conv_N) consisted of a convolutional layer, one layer of batch normalization, one layer of max pooling, and one layer of the Leaky ReLu function. The final output of the Deep Neural Network was a 5 × 1 vector. It was then passed through a softmax layer and finally outputted the predicted class (one of the five sleep stages). For the raw signals, the inputs of our CNN_LSTM were 30-second raw signal data of 4 channels (two EEG and two EOG) with an input size of 6000 × 4. The kernel layer was composed of two convolutional cells and one LSTM cell. Most of the set-ups were the same as those of the spectrogram CNN architecture, except structure, while the learning rate of ADAM was 0.001 and the batch size was set to 128.

## 3. Results and Discussion

### 3.1. Experimental Setup

In this section, we elaborate on the details of our experimental set-ups, outcomes, and the significance of the results. Figure 2 summarizes the overview of the automatic sleep stage classification process using a CNN model we developed. To evaluate the performance of the proposed CNN architecture, we designed two experiments: (1) training a CNN model that could correctly classify sleep stages and evaluate the performance with the ISRUC dataset, and (2) Using the trained CNN model to classify the sleep stages in the newly measured lab data. These two experiments were conducted on a laptop equipped with an Intel i7 processor (I7-9750H). To compare the transferability, both experiments were performed with two different types of input data: raw data and multi-taper spectrogram data.

In the first experiment, the first 100 subjects’ data were selected in subgroup 1 of the ISRUC dataset. To enhance the accuracy and minimize the bias associated with our classification model, only the epochs where the two scorers agreed with each other were used. The epochs were then split into three parts: 60% of the dataset for training (42,094 epochs), 20% for validation (14,032 epochs), and 20% for the test (14,032 epochs). The number of epochs of each of the five classes for the three separate parts is listed in Table 1. For each training step, the weight of the CNN network parameters was updated based on the result of model training validation accuracy. Most of the hyperparameter values (learning rate, kernel size, and filter of each convolutional layer, and unit of each dropout) were selected by a random search method. In the end, we chose the model with the highest validation accuracy as our best model. The performance of this best model was evaluated based on the prediction accuracy of the test dataset.

As a result, for the multi-taper spectrogram, CNN architecture, a (2,2) pool size of 2D-max pooling, 64 filters and a (3,3) kernel size were used on the first two 2D-convolutional layers and 32 filters and a (3,3) kernel size were used on the last 2D-convolutional layers. Moreover, in order, dense layers with the units 1024, 512 and 5 were used for the fully-connected layer. Batch normalization, first dropout (0.25), and second dropout (0.40) were utilized to prevent overfitting. For the raw-signal CNN + LSTM architecture, four pool sizes of 1D-max pooling, 80 filters, and three kernel sizes were used on the first 1D convolutional layers, and 32 filters and five kernel sizes were used on the last 1D-convolutional layers and unit 10 on the LSTM layers. Moreover, in order, dense layers with the units 64 and 5 were used for the fully-connected layer. Batch normalization and dropout (0.45) were utilized to prevent overfitting (Figure 2C,D).

In the second experiment, we evaluated the transferability of our trained CNN model on our lab dataset. We used the same model architecture as the first experiment. Our best model was used to predict sleep stages in the lab dataset, and this prediction performance was evaluated through comparison with the manual sleep stage scorings performed, based on AASM criteria, by one human expert with about one year of experience.

### 3.2. Performance Comparison with Other Works

**Figure 3:**
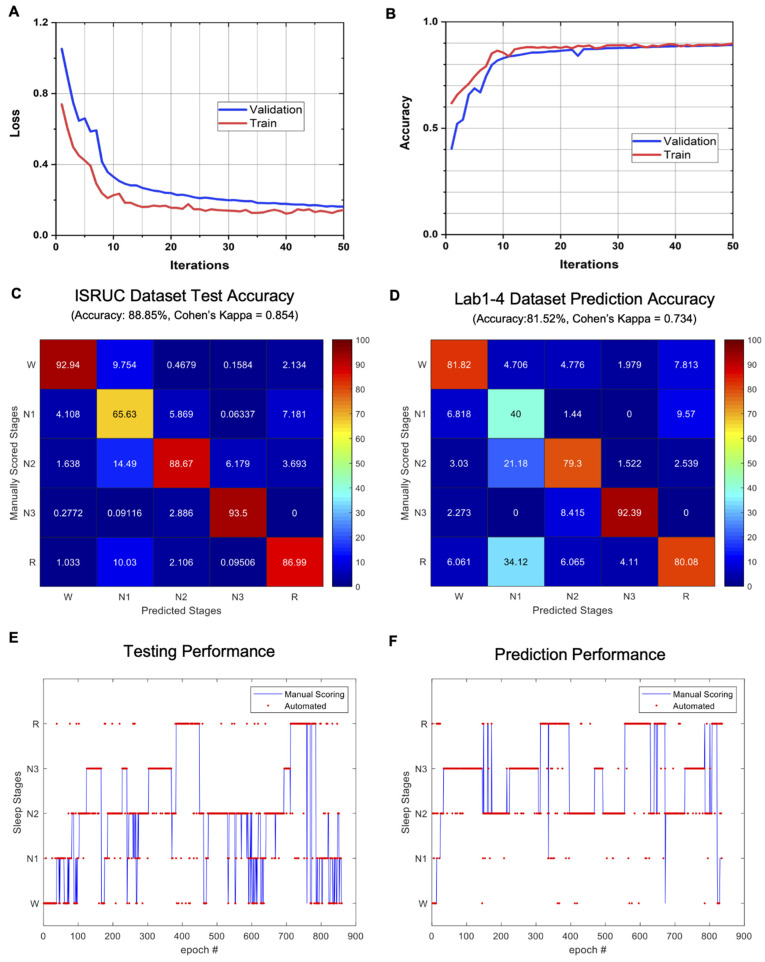
CNN classification results. (**A**) Loss curves of the training on the ISRUC dataset (red: loss on the training; blue: loss on the testing). (**B**) Accuracy curves of the training on the ISRUC dataset (red: accuracy on the training; blue: accuracy on the testing). (**C**) Confusion matrix with the public dataset (accuracy 88.85%, Cohen’s kappa = 0.854). (**D**) Confusion matrix with lab dataset (accuracy 81.52%, Cohen’s kappa = 0.734). (**E**,**F**) Hypnograms of prediction results from the ISRUC (**E**) and lab datasets (**F**).

The classification model was well-trained, based on the accuracy and loss of the training and validation graphs shown in Figure 3A,B. Table 2 summarizes the result of the first experiment. As a result of the first experiment with multi-taper spectrogram input data, our classification model’s prediction of the 100 subjects showed 88.85% accuracy and a Cohen’s kappa value of 0.854 with the consensus scores of the two expert scorers of the ISRUC dataset (Figure 3C). The classification results with raw data as input showed an accuracy of 87.05% and a Cohen’s kappa value of 0.829.

Table 3 summarizes the result of the second experiment and enumerates the number of epochs of each sleep stage that were included in the lab dataset. As a result of the second experiment with the multi-taper spectrogram input data, an accuracy of 81.85% and a Cohen’s kappa value of 0.734 could be achieved when the exact same classification model was used to predict the lab dataset measured with our own system (Figure 3D). The classification results with raw data as input showed an accuracy of 72.94% and a Cohen’s kappa value of 0.608. The results of these experiments clearly show that, compared to using just raw signal, converting the signal to multi-taper spectrogram as the input data provides not only comparable or higher classification performance within the public dataset but also superior transferability of the trained model for the classification of another dataset. The average inter-scorer agreement on standard PSG data was usually reported between 82% and 89% [3]. In agreement with this reported value, the average agreement between the two expert scorers of the ISRUC dataset was calculated to be 82.00%, with a Cohen’s kappa value of 0.766. The results of the second experiment (81.85%) fall very close to this expected inter-scorer reliability value, and this can show the potential of the effective transferability of our classification model into our lab-based custom system with high performance. The hypnograms in Figure 3E,F show the prediction results from the ISRUC and lab datasets, respectively.

Table 4 compares the performance of our CNN method in sleep stage classification with other prior works. To evaluate the performance of the sleep stage classification, there are multiple performance metrics being used in the field, including sensitivity, specificity, and F-measure. Among these metrics, the accuracy rate and Cohen’s kappa coefficient are the most commonly used metrics [3], so these metrics are presented and used for comparison in this table. Most of the existing works focused on analyzing public sleep datasets, except for a few cases. Compared to the resulting accuracy and kappa values of these works, our work in the first experiment within the public dataset shows comparable performance. Among the prior works, the work from Bresch et al. presented a study similar to ours where a classification model was built from a public dataset (SIESTA) and transfer-tested to a private dataset [30]. Their results showed a kappa value of 0.760 in the public dataset and 0.703 in their private dataset. Compared to this work, our work shows improved performance with both the public and private datasets. Overall, this study shows the human-level performance of our CNN-based sleep classification model in scoring the standard PSG dataset and presents the potential of its effective transferability to other types of datasets, such as our own custom lab dataset with novel nanomembrane electrodes.

Although we could demonstrate transferability of the classification model from the public PSG dataset to a private dataset, the setup and results from the current study still possess some limitations that need to be considered for future studies. First, the public dataset used for this study, ISRUC subgroup 1, includes data from subjects with sleep disorders. Sleep patterns and signal characteristics of subjects with various sleep disorders are likely to be different from those of healthy subjects, and this aspect could have led to reduced performance when the classification model was applied to healthy subject data. Since this study was intended to build a model to classify data from healthy subjects, for future studies, the inclusion of data from a healthy population will be necessary and helpful to enhance the classification performance. Next, a small population of four subjects was used for the tested lab dataset to explore the potential of the transferability of the classification model and to compare the pre-processing methods for more effective transferability. To further validate the effectiveness of multi-taper spectrograms along with a CNN to build a more globally transferable classification model, a much larger number of subjects from various cohorts will need to be included in future studies.

Moreover, despite the transferability of the classification model shown in this study, there was a clear reduction in accuracy when the model was tested on the lab dataset. This reduction in the accuracy came from the intrinsic differences between the two different measurement settings, such as subject population, electrode type, equipment, sampling rate, etc. These discrepancies resulted in slightly different spectral analysis signal characteristics and reduced classification performance. One of the critical differences was the electrode locations, especially the EEG electrodes on the forehead and the common reference electrode on the nose. Although the nose is considered to be a relatively inactive area, nose referencing still suffers from larger artifacts from facial muscle activity in both the EEG and EOG channels, and especially EOG artifacts in the EEG channels, due to its proximity to the eyes [47,48] In our lab system, the EEG electrodes were placed closer to the eyes on the forehead, and the reference was placed on the nose. With the measurement and reference electrode of each EEG channel placed on the top and bottom sides of the eyes, the vertical eye movement signals observed in the EEG channels were large, and they were also larger than those observed in our EOG channels. As shown in Figure 1E, the described discrepancy in the signals observed from the EEG and EOG channels of each system could be observed, with slightly different spectral characteristics at each stage potentially leading to a reduction in accuracy, most likely in stages N1, where slow eye movement (SEM) is present, and R, where rapid eye movement (REM) is present. In stages N1 and R of the public dataset, the EOG channels generally showed stronger activity observed in the lower frequency range, caused by SEM and REM, as compared to the EEG channels. On the other hand, in the lab datasets, the EEG channels often show comparable or stronger activity in the lower frequency range compared to the EOG channels, caused by strong vertical eye movement signals in the EEG channels and stronger horizontal eye movement signals observed in the EOG channels. In addition to the well-known difficulty in classifying N1, the number of N1 epochs used for both training the model and prediction of the lab dataset was much smaller compared to other stages, and this could have resulted in the misrepresentation of the model’s classification performance of N1 with the numerical results obtained in this study [49,50]. These discrepancies between the systems and the smaller number of channels in our system could have led to less accurate scoring of the lab dataset, as it was not measured with the standard PSG setup used for proper scoring with AASM criteria, which resulted in larger uncertainty in the classification performance assessment. For future study, the use of both a standard setup and our novel lab-based setup is desired for more objective and fair classification performance assessment, comparison with expert scorings and inter-scorer reliability.

Furthermore, there were two factors that could have led to the overestimation of our classification model’s performance within the public dataset: (1) using consensus epochs for the testing of the model, and (2) using all subjects’ data throughout the training, validation, and testing without subject separation. Consensus epochs have clearer signal characteristics of corresponding sleep stages compared to the epochs without consensus, so using consensus epochs for the testing of model may have led to performance overestimation. For future studies, it would be preferred to use all epochs for performance evaluation, and compare the results with inter-scorer reliability. Moreover, due to signal characteristic variability from subject to subject, a more objective evaluation of the performance would be achieved by separating the epochs of certain subjects and keeping them independent for testing purposes alone. If the same subject’s epochs used for training and validation are also used for testing, the testing performance could be higher than the result obtained when the model is used on another subject’s data. For future studies, the use of the leave-one-subject-out method for the evaluation of the model would be preferred, for more objective evaluation and comparison with other works.

## 4. Conclusions

In this work, we presented an automatic sleep stage classification model that could achieve good performance on the public dataset and accurately predict the sleep stage on our own laboratory dataset. A set of nanomembrane electrodes and custom wireless circuits were used to record lab datasets with EEG and EOG from multiple subjects during their sleep. We developed a classification model based on CNN, which was utilized for training and validating the classifier model based on the ISRUC public dataset using two EEG (F3-A2, F4-A1) and two EOG (EOGL, EOGR) channels. Then we transferred our model to the classification of our experimental dataset, which was collected with the nanomembrane electrodes. Overall, the collective results show that our model had high performance on both the test dataset (accuracy = 88.85%, Cohen’s kappa = 0.854) and on our lab dataset (accuracy = 81.85%, Cohen’s kappa = 0.734). Future work will resolve the limitations of the current study discussed above and expand this research to include a larger group of sleep patients to measure data with the wearable system for automatic sleep stage classification.

## Figures and Tables

**Table 1:** Number of epochs of each sleep stage for training, validation, and testing.

Input Type	Number of Epochs (ISRUC Public Dataset)														
	Training Set					Validation Set					Test Set				
	Aw	N1	N2	N3	R	Aw	N1	N2	N3	R	Aw	N1	N2	N3	R
Raw signal	10,968	3299	13,366	8264	6197	3591	1148	4570	2692	2031	3617	1065	4492	2739	2119
Spectrogram	11,013	3275	13,210	8189	6207	3568	1073	4729	2675	1987	3575	1144	4469	2791	2053

**Table 2:** Public dataset based on classification models trained and tested with raw signals and multi-taper spectrograms.

Input Type	ISRUC Public Dataset	
	Test Accuracy	Cohen’s Kappa
Raw signal	87.05%	0.829
Multi-taper spectrogram	88.85%	0.854

**Table 3:** Lab dataset prediction based on classification models trained and tested with raw signal and multi-taper spectrograms, and the combined number of epochs of each sleep stage.

Input Type	Lab Dataset		Number of Epochs (Lab Dataset)				
	Prediction Accuracy	Cohen’s Kappa	Prediction Set				
			Aw	N1	N2	N3	R
Raw signal	72.94%	0.608	230	111	1091	721	554
Multi-taper spectrogram	81.52%	0.734	230	111	1091	721	554

**Table 4:** Comparison of sleep-stage classification performance with prior works.

Ref.	Year	Data Type	Input Data	Number of Subjects	Public Dataset	Private Dataset	Number of Channels	Classification Method
					Accuracy (%) /Kappa	Accuracy (%) /Kappa		
This work	2022	ISRUC and Lab dataset	Multi-taper spectrogram and Raw data	100	88.85/0.854 87.05/0.829	81.52/0.734 72.94/0.608	2 EEG, 2 EOG	CNN
[31]	1993	Private data	Extracted features	12	-	80.60/-	2 EEG, 1 EOG, 1 EMG	Multilayer Neural Network
[32]	2005	SIESTA	Extracted features	590	79.6/0.72	-	1 EEG, 2 EOG, 1 EMG	LDA, Decision tree
[5]	2014	Sleep-EDF	Extracted features	1	88.9/-	-	1 EEG	SVM
[33]	2016	Sleep-EDF	Raw data	20	74/0.65	-	1 EEG	CNN
[34]	2016	Sleep-EDF	Extracted features	20	78/-	-	1 EEG	Stacked Sparse Autoencoders
[35]	2017	Montreal archive	Extracted features	62	83.35/-	-	1 EEG	Mixed Neural Network
[36]	2017	Sleep-EDF & Montreal	Raw data	32	86.2/0.80	-	1 EEG	DeepSleepNET (CNN + LSTM)
[37]	2018	Montreal archive	Raw data	61	78/0.80	-	6 EEG, 2 EOG, 3 EMG	Multivariate Network
[38]	2018	Private dataset	Extracted features	76	-	-/0.8	1 EEG, 2 EOG	Random Forest, CNN, LSTM
[39]	2018	SHHS	Raw data	5728	87/0.81	-	1 EEG	CNN
[40]	2018	12 sleep centers	Raw data	1086	87/0.766	-	4 EEG, 2 EOG, 1 EMG	CNN
[7]	2018	ISRUC	Extracted features	100	75.29/-	-	6 EEG	Random Forest
[41]	2018	ISRUC	Raw data	116	92.2/-	-	6 EEG, 2 EOG, 3 EMG	CNN
[30]	2018	SIESTA/private data	Raw data	147	-/0.760	-/0.703	1 EEG, 2 EOG	RNN
[6]	2019	ISRUC	Extracted features	10	79.64/0.74	-	6 EEG	HMM
[42]	2019	Sleep-EDF	Raw data	61	91.22/-	-	1 EEG, 1 EOG	CNN
[43]	2019	Montreal archive	Extracted features	200	83.6/-	-	1 EEG, 1EOG, 1EMG	CNN
[44]	2020	ISRUC	Extracted features	10	81.65/0.76	-	1 EEG	IMBEFs
[45]	2020	Sleep-EDF	Raw data	100	85.52/-	-	2 EEG	CNN
[46]	2020	ISRUC	Raw data	294	81.8/0.72	-	2 EEG, 2 EOG, 1 EMG, 1 ECG	CNN + RNN

## Data Availability

The data presented in this study are available on request from the corresponding author.
